# Plasma NfL and GFAP in the preclinical stages of neurodegenerative diseases: insights from the UK Biobank

**DOI:** 10.1007/s00415-025-13498-y

**Published:** 2025-11-09

**Authors:** Jolanda Buonocore, Enrico Fratto, Fulvia Arcuri, Basilio Vescio, Camilla Calomino, Mariagrazia Talarico, Costanza Maria Cristiani, Aldo Quattrone, Andrea Quattrone

**Affiliations:** 1 Institute of Neurology, Department of Medical and Surgical Sciences, Magna Graecia University, Catanzaro, Italy; 2 Neuroscience Research Center, Magna Graecia University, Catanzaro, Italy; 3 Institute of Bioimaging and Complex Biological Systems, IBSBC-CNR, Catanzaro, Italy; 4 Laboratory of Stem Cells, Department of Medical and Surgical Sciences, Magna Graecia University, Catanzaro, Italy

**Keywords:** Neurofilament light chain, Glial fibrillary acidic protein, UK Biobank, Neurodegenerative diseases, Plasma biomarkers

## Abstract

**Background:**

Plasma neurofilament light chain (NfL) and glial fibrillary acidic protein (GFAP) are promising blood-based biomarkers of neuroaxonal injury and astrocytic activation, relevant in neurodegeneration. We investigated their pre-diagnostic profiles across common neurodegenerative diseases, including Parkinson’s disease (PD), atypical parkinsonian disorders (APD), Alzheimer’s disease (AD), and amyotrophic lateral sclerosis (ALS).

**Methods:**

Forty-eight thousand five hundred and twenty-four UK Biobank participants with baseline plasma proteomic data for NfL and GFAP were included. Incident diagnoses of PD, APD, AD, and ALS were identified through ICD-10—coded health records, after careful inclusion/exclusion procedures. Baseline plasma NfL and GFAP concentrations were standardized as *z*-scores adjusted for relevant covariates. The hazard ratio (HR) for incident neurodegenerative diseases was estimated using Cox regression models adjusted for demographic, clinical, socioeconomic and genetic factors.

**Results:**

The final sample included 1196 cases (505 PD, 26 APD, 476 AD, 189 ALS) and 44,107 control subjects. In Cox regression models, NfL was associated with higher risk of incident ALS (HR 1.69; 95% CI, 1.58–1.80, *p* < 0.001), APD (HR 1.51; 95% CI, 1.22–1.87, *p* < 0.001), AD (HR 1.31; 95% CI, 1.22–1.41, *p* < 0.001), and PD (HR 1.14; 95% CI, 1.05–1.23, *p* < 0.001). GFAP was independently associated with incident AD only (HR 1.72; 95% CI, 1.63–1.82, *p* < 0.001).

**Conclusion:**

Plasma NfL and GFAP showed distinct pre-diagnostic profiles. NfL was associated with incident diagnosis of several neurodegenerative diseases, while GFAP was specific to AD, reinforcing its role in dementia. These results may help optimize the identification of target populations at risk of neurodegenerative diseases for future neuroprotective treatments.

**Supplementary Information:**

The online version contains supplementary material available at 10.1007/s00415-025-13498-y.

## Introduction

Neurodegenerative diseases represent an increasing social and economic burden, particularly in the context of a globally aging population [1, 2]. Despite growing awareness and intensified research efforts, diagnostic accuracy remains suboptimal across different levels of clinical care. In this context, there is an urgent need for early, non-invasive biomarkers that can enhance diagnostic accuracy, guide prognosis, and inform therapeutic strategies [2–4], especially with the advent of disease-modifying therapies.

NfL and GFAP, among other potential biomarkers, have emerged as promising indicators of neurodegeneration and neuroinflammation, respectively [3]. The possibility to be measured in cerebrospinal fluid (CSF) and blood-based matrices, with strong inter-fluid correlations and robust associations with imaging and clinical outcomes [5, 6], offers hope for improved diagnosis and treatment of neurodegenerative diseases.

NfL is a structural axonal protein released in response to neuronal injury, elevated across a broad spectrum of neurodegenerative disorders, including multiple sclerosis, dementia, amyotrophic lateral sclerosis (ALS), and atypical parkinsonian disorders (APD) [7, 8]. Beyond its role in differential diagnosis [9–12], NfL is now recognized as a dynamic marker of neuronal damage, reflecting the intensity of neurodegenerative processes and responding to therapeutic interventions [13–16]. Notably, evidence from prodromal disease cohorts suggests that NfL levels begin to rise years before clinical onset in several disorders: up to 12–24 months in ALS [17] and even earlier in AD and Parkinson’s disease (PD) [18–20].

On the other hand, GFAP is an intermediate filament protein expressed by astrocytes, and its elevation reflects reactive astrogliosis, a process tightly linked to neuroinflammation. Elevated plasma GFAP has been recently authorized by the FDA for clinical use in mild traumatic brain injury [21] and has received growing attention in recent years in the neurodegenerative field. It has been consistently associated with the development and progression of cognitive impairment [22, 23], especially related to Alzheimer’s-type pathology [24]. However, increased GFAP levels have also been found in patients with PD, APD, frontotemporal dementia (FTD), multiple sclerosis, prion diseases, and ALS [25–31]. GFAP elevation has also been observed in the preclinical phase of AD [24, 32], and a large-scale study from the UK Biobank recently demonstrated that higher plasma levels of NfL and GFAP were associated with increased risk of all-cause dementia, including AD, FTD, and vascular dementia [33]. However, despite growing interest in their pre-diagnostic value, direct comparative studies of NfL and GFAP across neurodegenerative diseases remain scarce, making their temporal trajectories and disorder-specific predictive potential not fully understood.

In the current study, we leveraged the prospective cohort from the UK Biobank to carry out a large-scale proteomic data analysis from nearly 50,000 participants. Our primary aim was to examine and compare plasma NfL and GFAP levels in the pre-diagnostic phase of multiple neurodegenerative diseases, including Alzheimer’s disease, parkinsonian syndromes, and ALS, investigating their associations with each other and with incident neurodegenerative diseases.

## Methods

### Participants

**Fig. 1:**
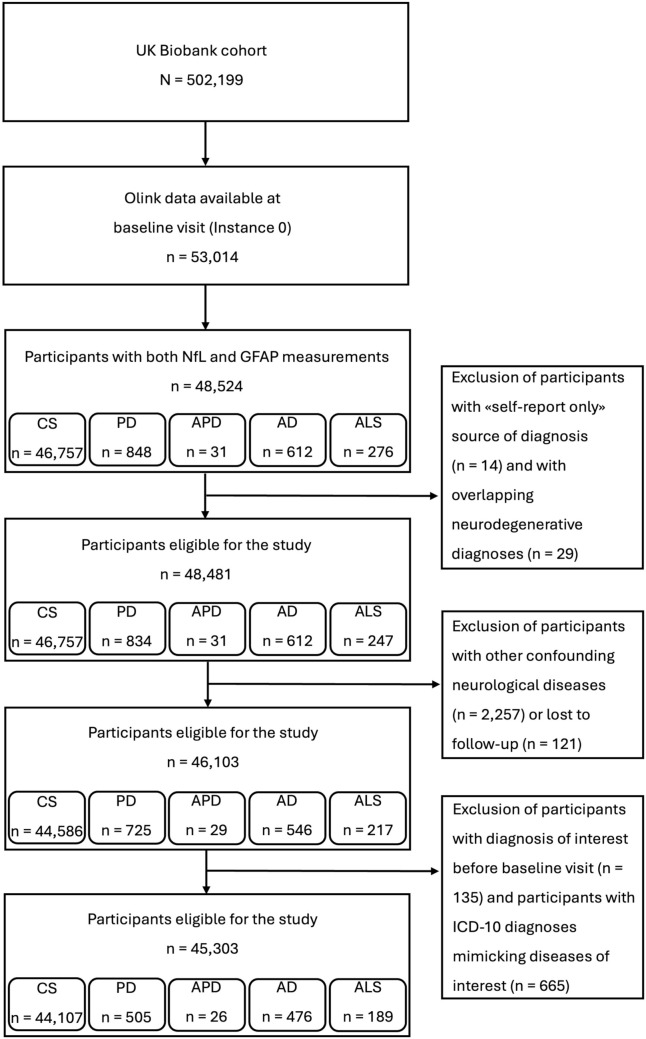
Participant selection flowchart for the study. This diagram illustrates the sequential exclusion of participants from the UK Biobank cohort based on predefined criteria. Details are provided in Supplementary Materials and Supplementary Tables 1–2. *CS* Control subjects, *PD* Parkinson’s disease, *APD* Atypical parkinsonian disorders, *AD* Alzheimer’s disease, *ALS* Amyotrophic lateral sclerosis, *NfL* Neurofilament light chain, *GFAP* Glial fibrillary acidic protein

This study utilized data from the UK Biobank, a large prospective cohort of approximately 500,000 participants registered with the UK National Health Service and recruited from 22 assessment centers between 2006 and 2010. In the present study, we focused on five major neurodegenerative conditions, hereafter referred to as “diagnoses of interest”: PD, APD (including progressive supranuclear palsy [PSP] and multiple system atrophy [MSA]), AD, and ALS. Diagnoses were identified using ICD-10 codes from linked health records. Inclusion criteria were: (a) availability of both baseline plasma NfL and GFAP measurements obtained using the Olink Explore 3072 platform; and (b) incident diagnosis of interest during the observational period (for patient groups) or absence of any neurodegenerative condition throughout follow-up (for control subjects, CS). Within the control group, a subset of healthy controls (HC) was defined as participants with no ICD-10 diagnoses of any kind, either neurological or non-neurological. Exclusion criteria, applied to both patients and controls, were as follows: (a) low diagnostic certainty, defined as diagnoses based solely on self-reported data (“self-report only”); (b) presence of neurological conditions that may act as confounders or mimic the diseases of interest (see Supplementary Materials and Supplementary Table 1); (c) loss to follow-up; and (d) diagnosis of interest already present at baseline visit (Field ID 53: “Date of attending assessment center”). A study flowchart is shown in Fig. 1.

Incident disease group classification based on ICD-10 diagnoses was performed according to carefully selected inclusion/exclusion criteria based upon clinical reasoning established by a consensus among three authors with clinical expertise in movement disorders and dementia. A detailed description is provided in the “Health-related outcomes” section in the Supplementary Materials and Supplementary Tables 1–2. The mean observation time, defined as the interval from baseline assessment to the earliest event among death, loss to follow-up, or administrative censoring (30 November 2022), was 13.36 ± 2.09 years. All participants provided informed consent for data collection, long-term health record linkage, and use of their data for research purposes.

### Plasma NfL and GFAP levels

The plasma NfL and GFAP levels were measured through the Olink Explore 3072 platform, which uses Proximity Extension Assay combined with Next-Generation Sequencing technology to measure approximately 3000 proteins in biological samples. Blood samples were collected from all study participants at the baseline visit. The serum was prepared and stored at −80 °C until assay. Detailed assay procedures and quality control measures are available at https://biobank.ndph.ox.ac.uk/ukb/ukb/docs/Olink_proteomics_data.pdf. Plasma NfL and GFAP levels were available in Normalized Protein eXpression (NPX) units on a log₂ scale, according to the Olink standard output.

### Statistical analysis

All analyses were conducted using R (v2024.12.0.467). Continuous variables are presented as means and standard deviations, while categorical variables as counts and percentages. Raw values of baseline NfL, GFAP, cognitive scores (fluid intelligence and reaction time), and hand grip strength were converted into *w*-scores (*z*-scores adjusted for relevant covariates) using linear regression models based on the HC group with no ICD-10 diagnoses (*n* = 4926), as described previously and in Supplementary Materials. Specifically, covariates included age, sex, and BMI for NfL, GFAP and grip strength; age, sex, and education for the cognitive measures.

Group comparisons were performed using ANOVA, ANCOVA, or Kruskal–Wallis tests, followed by Bonferroni-corrected post hoc tests. Categorical variables were compared using chi-squared tests. Given the large and imbalanced samples, effect sizes (Cohen’s *d* for continuous variables and Cramér’s V for categorical ones) were reported alongside *p*-values. Spearman’s rank correlations assessed associations between biomarkers and cognitive or motor performance. The relationship between baseline NfL and GFAP levels and subsequent risk of neurodegenerative diseases was examined using Cox proportional hazards models, with hazard ratios (HRs) and 95% confidence intervals (CIs) estimated across six adjusted models. The proportional hazards assumption was formally tested using Schoenfeld residuals. For models where this assumption was violated, we incorporated time-dependent effects by including interactions with log(time), allowing HRs to vary over follow-up. Time intervals between blood sampling and diagnosis were expressed as years before diagnosis and annual mean *w*-scores were computed. Linear regression models were then applied to assess whether levels varied as the diagnosis approached. All analyses are detailed in Supplementary Materials.

## Results

### Population characteristics

The final cohort comprised 45,303 participants (Fig. 1). Among them, 1196 developed a neurodegenerative disease during the follow-up, including 505 developing PD, 26 APD (14 PSP and 12 MSA), 476 AD, and 189 ALS. The remaining 44,107 participants were free of these diagnoses at the last follow-up and served as CS.

At baseline, CS were generally younger than the other groups, and incident AD subjects included a higher proportion of women. Incident AD cases had a lower prevalence of high-degree education and alcohol consumption than other groups, while smoking prevalence was significantly lower in both incident PD and AD groups. Vascular and metabolic comorbidities (type 2 diabetes and hypertension) were more frequent in disease groups, particularly in incident PD, AD, and APD. Genetic data reflected disease-specific patterns: APOE ε4 carriers were more common in the incident AD group, while family history of PD or dementia was more frequent in incident PD and AD groups, respectively. These associations were further supported by polygenic risk scores (PRS), which were elevated in the relative disease groups. At baseline, hand grip strength (motor function and overall physical health) was significantly reduced in both incident PD and AD cases; moreover, reduced fluid intelligence (reflecting reasoning and problem-solving abilities) and increased reaction time (processing speed and motor-cognitive integration) were observed in subjects with a future incident diagnosis of AD compared to controls. Small effect sizes in these variables compared to CS were also evident for APD and ALS, though the comparisons did not reach statistical significance. No significant correlations after FDR were observed between blood protein levels and these clinical features at baseline.

### NfL and GFAP comparisons across groups

**Fig. 2:**
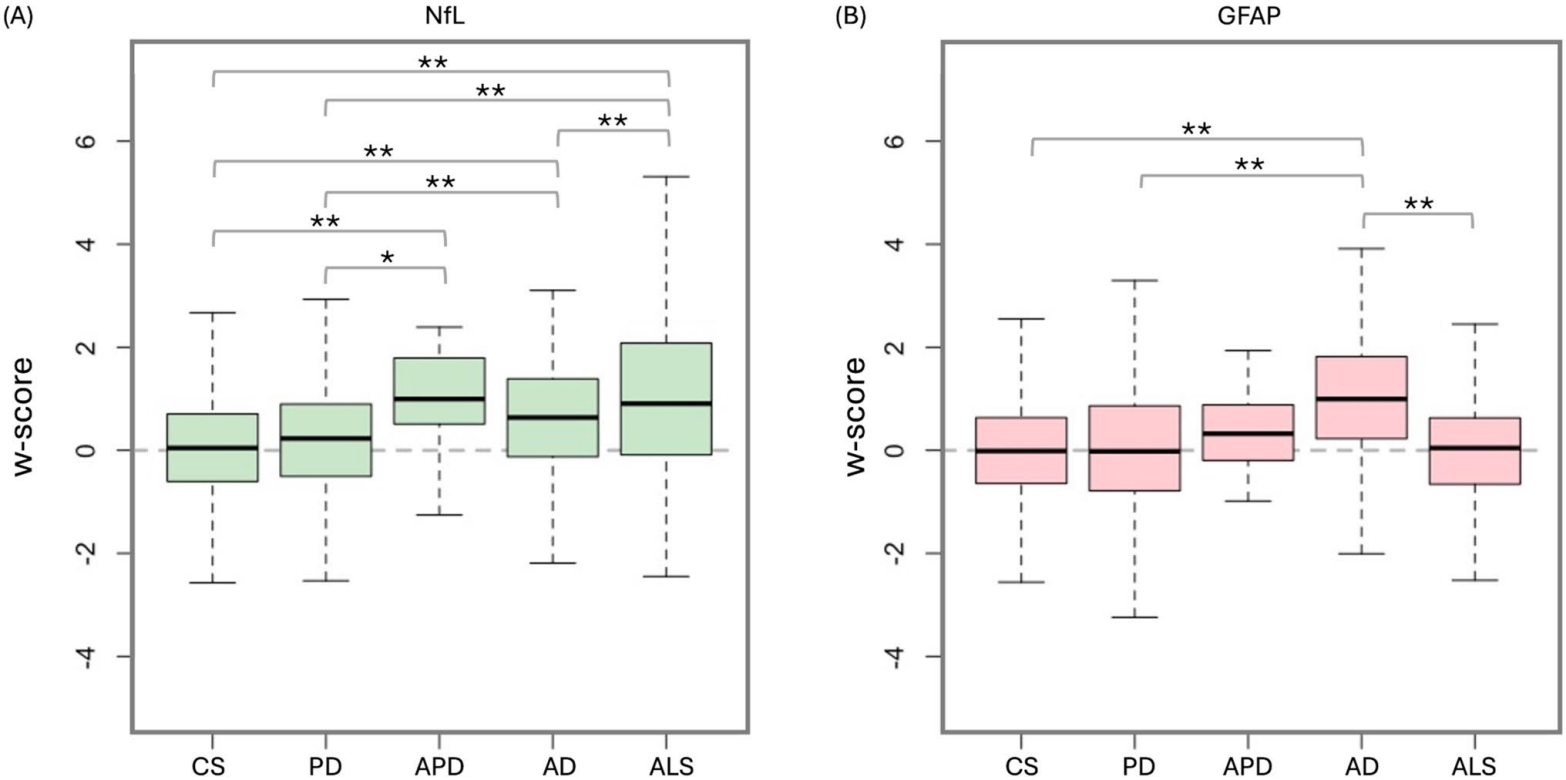
Group-wise distribution of NfL and GFAP w-scores at baseline. (**A**) Boxplot of NfL *w*-scores across diagnostic groups, showing a progressive increase from CS to individuals who later developed PD, AD, APD and ALS. (**B**) Boxplot of GFAP *w*-scores, demonstrating a marked elevation in individuals later diagnosed with AD compared to all other groups. Horizontal brackets indicate statistically significant differences from post hoc comparisons, after ANCOVA with eGFR and time from blood collection to diagnosis (*p < 0.05; **p < 0.001). Outliers are not shown in the plots for visual representation. *CS* Control subjects, *PD* Parkinson’s disease, *APD* Atypical parkinsonian disorders, *AD* Alzheimer’s disease, *ALS* Amyotrophic lateral sclerosis, *NfL* Neurofilament light chain, *GFAP* Glial fibrillary acidic protein

At baseline, all subjects were free of any clinical diagnosis of interest. However, NfL *w*-scores were elevated in individuals who later developed ALS, APD, AD, or PD in this descending order (Table 1). ALS and APD showed large elevations versus CS and PD; AD showed moderate increases, while PD differed minimally from CS (Fig. 2A). In contrast, GFAP *w*-scores were selectively elevated in individuals who later developed AD, with no substantial increases in PD, APD, or ALS (Fig. 2B). All analyses were adjusted for eGFR. These findings were overall consistent using either *w*-scores or raw NPX values (Supplementary Fig. 1), although raw values showed slightly more marked differences. Notably, repeating the analyses while adjusting for the time interval between biomarker assessment and clinical diagnosis confirmed the robustness of the findings.

**Table 1:** Baseline demographics and clinical characteristics of study participants across groups

Data	CS (*n* = 44,107)	PD (*n* = 505)	APD (*n* = 26)	AD (*n* = 476)	ALS (*n* = 189)	*P* value	Post-hoc Significant *p* value	Effect size (≥0.2)**
Sex (% Female)	24,032 (54.5)	194 (38.4)	15 (57.7)	294 (61.8)	99 (52.4)	** <0.001**^**a**^	CS > PD AD > CS, PD, ALS ALS > PD	AD > PD ALS > PD
Age at baseline visit, years^*^	56.9 (8.2)	63.2 (5.2)	62.8 (5.7)	65.6 (5.0)	60.5 (6.9)	** <0.001**^**b**^	PD > CS, ALS APD > CS AD > CS, PD, ALS ALS > CS	PD > CS, ALS APD > CS, ALS AD > CS, PD, APD, ALS ALS > CS
Age at diagnosis onset, years^*^	–	69.1 (6.1)	69.9 (7.9)	74.9 (6.1)	66.3 (7.6)	** <0.001**^**c**^	PD > ALS APD > ALS AD > PD, APD, ALS	PD > ALS APD > ALS AD > PD, APD, ALS
Time to diagnosis, years^*^	–	5.9 (3.7)	7.1 (4.5)	9.3 (3.3)	5.9 (3.3)	** <0.001**^**c**^	AD > PD, APD, ALS	APD > PD, ALS AD > PD, APD, ALS
Observation time, years^*^	13.4 (2.0)	12.3 (2.9)	10.1 (3.3)	12.6 (2.3)	7.9 (3.8)	** <0.001**^**b**^	CS > PD, APD, AD, ALS PD > APD, ALS AD > APD, ALS	CS > PD, APD, AD, ALS PD > APD, ALS AD > APD, ALS
Socioeconomic								
Townsend Deprivation Index^*^	−1.2 (3.2)	−1.5 (2.9)	−1.4 (3.3)	−1.8 (3.3)	−1.3 (2.9)	0.45^c^	–	–
Ethnicity (% White)	41,261 (93.6)	486 (96.2)	24 (92.3)	456 (95.8)	184 (97.4)	** <0.05**^**a**^	–	–
College or University degree (%)	14,348 (32.5)	154 (30.5)	4 (15.4)	105 (22.1)	51 (27.0)	** <0.001**^**a**^	CS > AD PD > AD	–
Lifestyle factors								
Current smokers (%)	4630 (10.5)	19 (3.8)	3 (11.5)	33 (6.9)	21 (11.1)	** <0.001**^**a**^	CS > PD ALS > PD	ALS > PD
Current alcohol (%)	40,408 (91.6)	451 (89.3)	25 (96.2)	402 (84.5)	172 (91.0)	** <0.001**^**a**^	CS > AD	–
Comorbidities								
BMI^*^	27.4 (4.8)	27.4 (4.4)	28.9 (4.9)	27.3 (4.8)	27.5 (4.7)	0.42^b^	–	APD > CS, PD, AD, ALS
Diabetes type 2 (%)	3912 (8.9)	90 (17.8)	6 (23.1)	93 (19.5)	23 (12.2)	** <0.001**^**a**^	AD > CS PD > CS	–
Hypertension (%)	14,065 (31.9)	294 (58.2)	15 (57.7)	274 (57.6)	77 (40.7)	** <0.001**^**a**^	AD > CS, ALS PD > CS, ALS	AD > ALS PD > ALS
eGFR^*^	103.1 (17.8)	95.9 (16.0)	96.6 (21.7)	94.3 (17.9)	100.7 (17.6)	** <0.001**^**b**^	CS > PD, AD ALS > PD, AD	CS > PD, APD, AD ALS > PD, APD, AD
Genetic factors								
PD family history (%)	1734 (3.9)	50 (9.9)	3 (11.5)	27 (5.7)	8 (4.2)	** <0.001**^**a**^	PD > CS	–
Family history of AD/dementia (%)	5033 (11.4)	86 (17.0)	4 (15.4)	121 (25.4)	34 (18.0)	** <0.001**^**a**^	AD > CS, PD PD > CS	AD > PD
PRS for PD^*^	−0.1 (1.0)	0.3 (1.1)	−0.1 (0.9)	−0.1 (1.0)	−0.3 (1.1)	** <0.001**^**c**^	PD > CS, AD, ALS	PD > CS, APD, AD, ALS
PRS for AD^*^	0.04 (1.0)	0.04 (1.1)	0.05 (1.0)	1.2 (1.3)	0.04 (1.0)	** <0.001**^**b**^	AD > CS, PD, APD, ALS	AD > CS, PD, APD, ALS
ApoE-ε4 carrier (%)	11,861 (26.9)	127 (25.2)	4 (15.4)	333 (70.0)	50 (26.5)	** <0.001**^**a**^	AD > CS, PD, APD, ALS	AD > PD, APD, ALS
Pre-diagnostic cognitive and motor function								
Fluid intelligence^*,#^	−0.1 (1.0)	−0.3 (1.0)	−0.4 (0.8)	−0.4 (0.8)	−0.5 (1.3)	** <0.001**^**a**^	CS > AD	CS > APD, AD, ALS PD > AD
Reaction time^*,#^	−0.01 (1.0)	0.03 (1.0)	0.2 (1.1)	0.2 (1.2)	−0.02 (0.9)	** <0.05**^**a**^	AD > CS	APD > CS, ALS AD > CS, PD, ALS
Hand grip^*,#^	−0.1 (1.0)	−0.4 (1.1)	−0.4 (0.7)	−0.4 (0.9)	−0.3 (1.1)	** <0.001**^**a**^	CS > PD, AD	CS > PD, APD, AD
Protein biomarker level								
NfL^*,#^	0.1 (1.1)	0.3 (1.3)	1.1 (1.2)	0.7 (1.1)	1.3 (2.0)	** <0.001**^**d**^	APD > CS, PD AD > CS, PD ALS > CS, PD, AD	APD > CS, PD AD > CS, PD, APD ALS > CS, PD, AD
GFAP^*,#^	0.007 (1.0)	0.01 (1.3)	0.4 (0.9)	1.0 (1.2)	0.07 (1.2)	** <0.001**^**d**^	AD > CS, PD, ALS	APD > CS, PD, ALS AD > CS, PD, APD, ALS

*CS* Control subjects, *PD* Parkinson’s disease, *APD* Atypical parkinsonian disorders, *AD* Alzheimer’s disease, *ALS* Amyotrophic lateral sclerosis, *BMI* Body mass index, *eGFR* Estimated glomerular filtration rate, *apoE-ε4* Apolipoprotein E-epsilon4, *PRS* Polygenic risk score, *NfL* Neurofilament light chain, *GFAP* Glial fibrillary acidic protein.

Information on APOE genotype, PRS, baseline motor/cognitive function and educational qualifications (specifically regarding “College or University degree” status) was available for a participant subgroup. Detailed information on missing data is provided in Supplementary Table 4. Family history of PD or AD/dementia refers to the presence of the respective condition in first-degree relatives (parents or siblings, see Supplementary Materials).

^*^Mean (standard deviation).

^**^We considered as meaningful effect sizes ≥0.2 (threshold for a small effect) in Cohen-D for continuous variables and Cramér’s *V* ≥ 0.1 for categorical variables.

^#^*w*-scores computed on the healthy controls with no ICD-10 diseases. Covariates included: age, sex, and BMI for NfL, GFAP and hand grip strength; age, sex and years of education for fluid intelligence (reasoning and problem-solving), reaction time (motor-cognitive processing).

^a^chi-squared test

^b^Kruskal–Wallis test

^c^ANCOVA with sex and age as covariates

^d^ANCOVA with estimated glomerular filtration rate (eGFR) as covariates

Significant *p* values are highlighted in bold

### Proportion and temporal trends of elevated NfL and GFAP levels across clinical groups

**Fig. 3:**
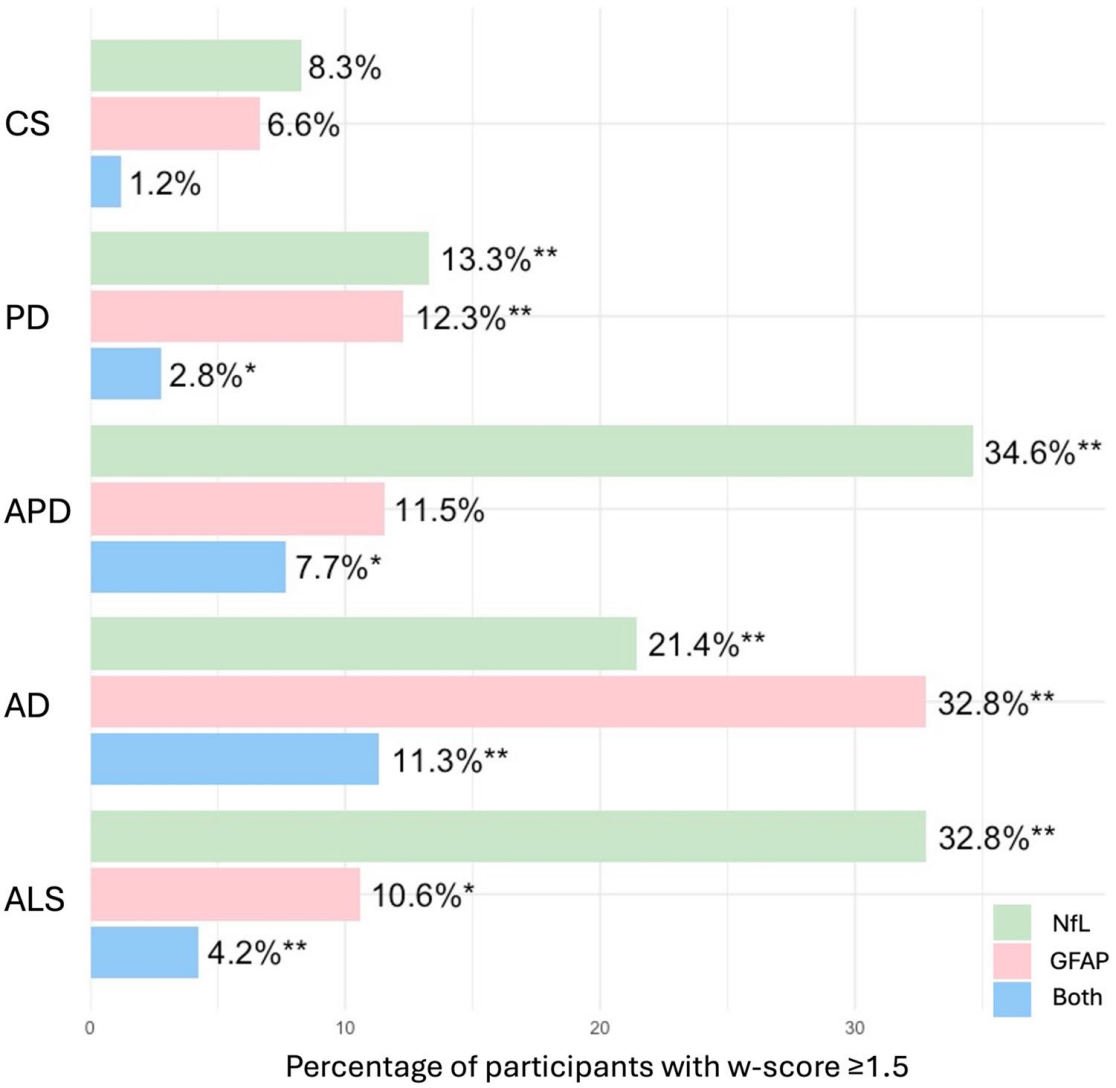
Proportion of individuals with elevated NfL and GFAP levels (defined as *w*-score ≥1.5) across diagnostic groups. Bar plots show the percentage of participants in each group with elevated levels of NfL, GFAP, or both. *CS* Control subjects, *PD* Parkinson’s disease, *APD* Atypical parkinsonian disorders, *AD* Alzheimer’s disease, *ALS* Amyotrophic lateral sclerosis, *NfL* Neurofilament light chain, *GFAP* Glial fibrillary acidic protein

We assessed the prevalence of elevated NfL and GFAP levels across clinical groups (Fig. 3), defining elevation as *w*-scores ≥1.5, consistent with previous studies [34, 35]. NfL elevation was most frequent in APD and ALS, followed by AD and PD, reflecting a gradient from more to less aggressive disorders. In contrast, GFAP increase showed a more selective profile in AD remaining rare in the other groups. Exploring the co-occurrence of elevated levels of both biomarkers, this scenario was rarely seen in CS (1.2%, 528/44,107) but was more common in disease groups, most notably in AD (11.3%, 54/476), followed by APD (7.7%, 2/26), ALS (4.2%, 8/189), and PD (2.8%, 14/505). The proportion of individuals with pathological levels of both biomarkers was significantly higher in all disease groups compared with controls (Chi-squared test, *p* < 0.001 for AD, and ALS; *p* < 0.05 for PD and APD).

**Fig. 4:**
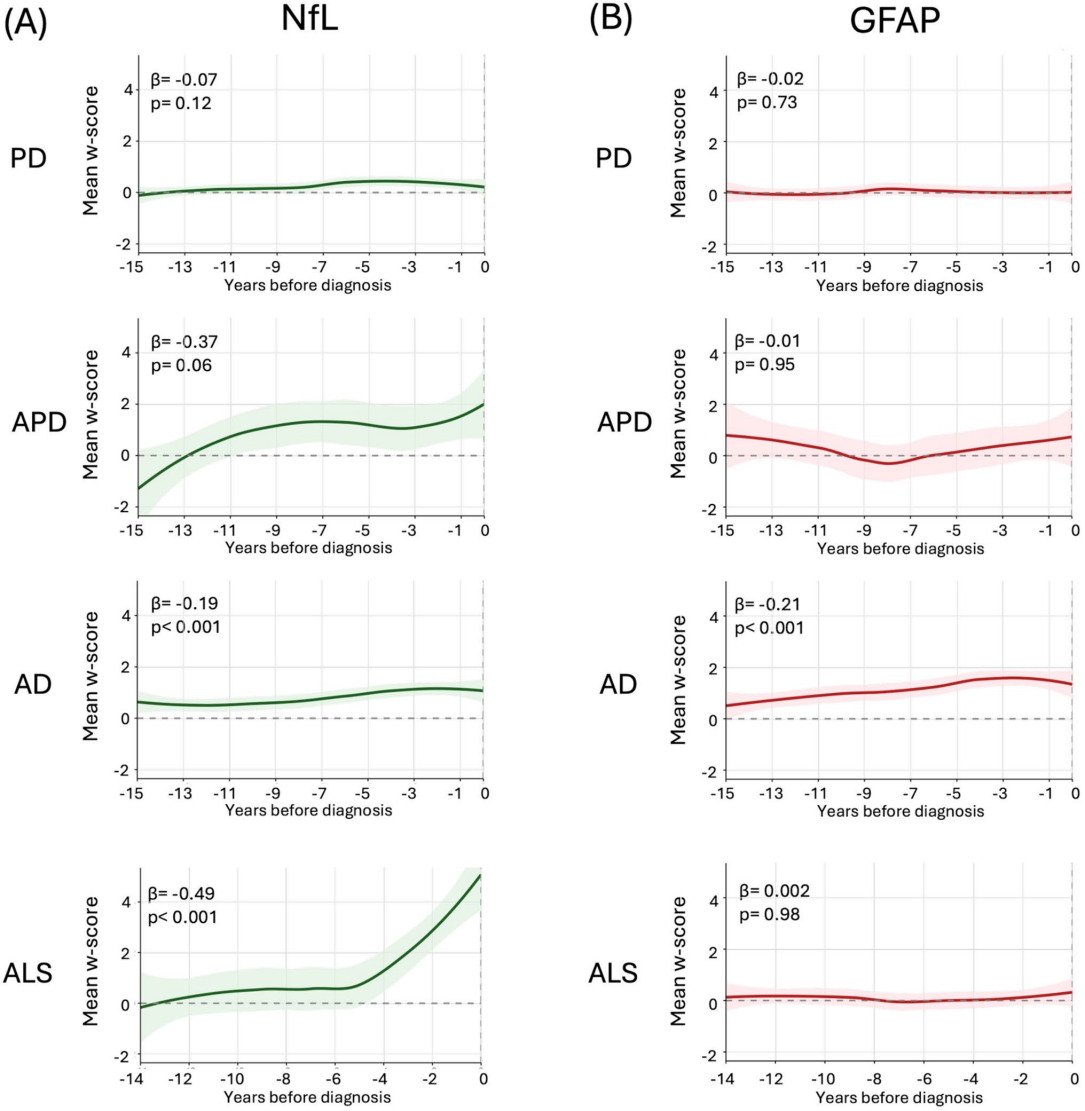
Trajectories of mean plasma NfL and GFAP *w*-scores in the years preceding clinical diagnosis across disease groups. Values represent annual means by diagnostic group and biomarker, aligned to the time of diagnosis (year 0). NfL levels exhibited a pre-diagnostic increase in all groups, especially ALS and APD. In contrast, GFAP displayed an upward trend only in AD. Shaded areas represent 95% confidence intervals around LOESS-smoothed curves. Reported *β* coefficients and *p*-values correspond to the slope and significance of the association between time (years before diagnosis) and biomarker levels. *CS* Control subjects, *PD* Parkinson’s disease, *APD* Atypical parkinsonian disorders, *AD* Alzheimer’s disease, *ALS* Amyotrophic lateral sclerosis, *NfL* Neurofilament light chain, *GFAP* Glial fibrillary acidic protein

To capture temporal dynamics, we reconstructed trajectories of plasma NfL and GFAP over the 15 years preceding diagnosis (Fig. 4). Slight NfL elevation was already evident 15 years before diagnosis in patients with AD and occurred in APD and ALS around 10 and 5 years before diagnosis, respectively, with the steepest increase in ALS and the less straightforward trend in PD. Significant associations between NfL values and time to diagnosis were observed in incident ALS (*β* regression coefficient: −0.49, *p* < 0.001) and AD (*β*: −0.19, *p* < 0.001), with a trend in atypical parkinsonism (*β*: −0.37, *p* = 0.06), In contrast, GFAP showed a progressive increase only in AD, peaking in the 5 years before diagnosis and showing significant association with time to diagnosis (*β*: −0.21, *p* < 0.001) while remaining flat in PD, APD, and ALS.

### Association between peripheral NfL and GFAP and risk of neurodegenerative diseases

**Fig. 5:**
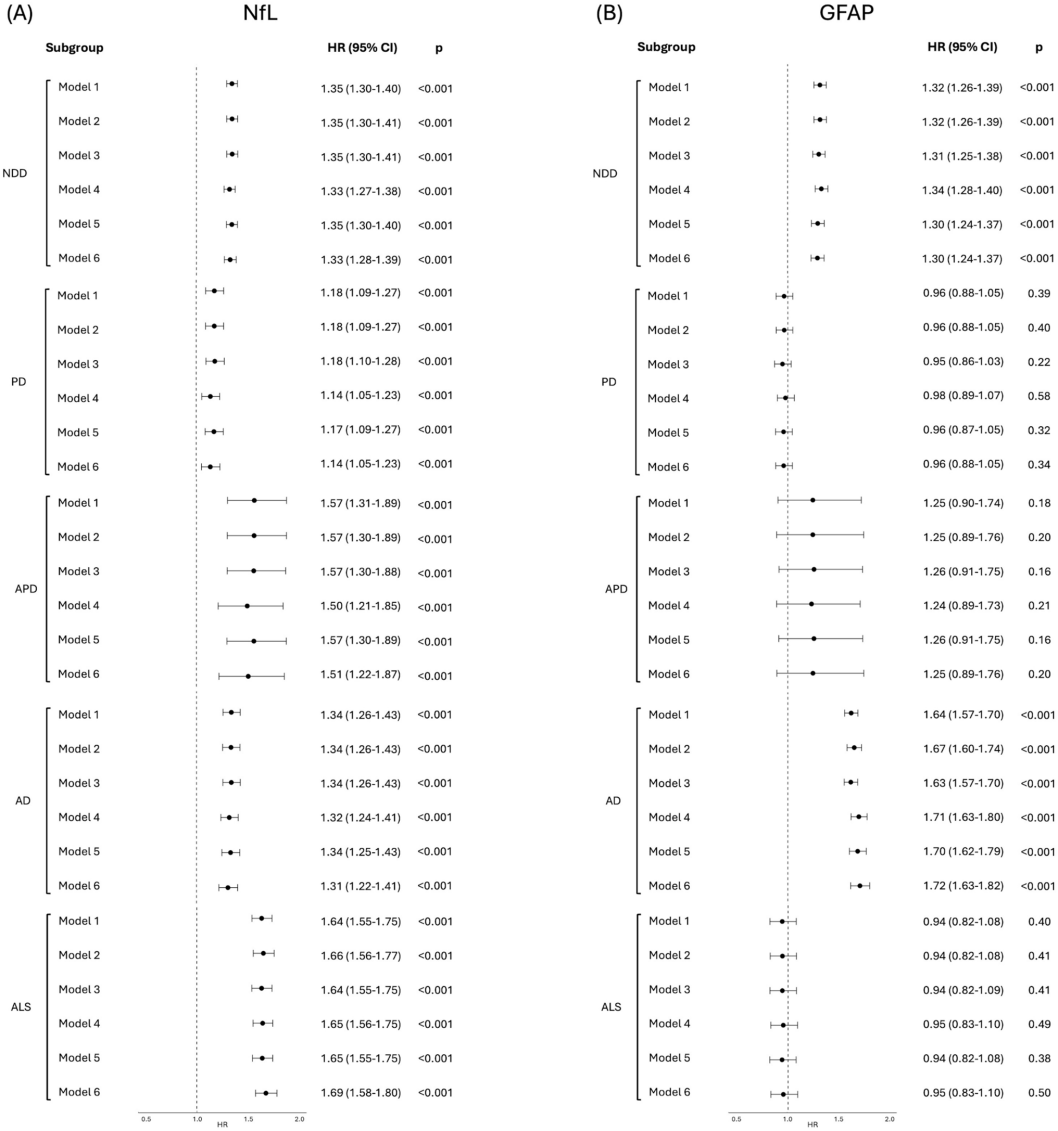
Hazard ratios for the independent association of NfL and GFAP with incident neurodegenerative diseases. (**A**) Forest plot showing hazard ratios (HRs) and 95% confidence intervals (CIs) for NfL across six adjustment models in any one of the neurodegenerative diseases of interest, as well as each diagnosis separately. (**B**) Forest plot showing the corresponding HRs and CIs for GFAP across the same models and diagnostic groups. Each HR estimate was derived from Cox proportional hazards models, adjusted for relevant covariates as described in the Supplementary Materials. The dashed vertical line is at HR = 1 (no association). *HR* Hazard ratio, *CI* Confidence interval, *NfL* Neurofilament light chain, *GFAP* Glial fibrillary acidic protein, *NDD* Neurodegenerative diseases, *PD* Parkinson’s disease, *APD* Atypical parkinsonian disorders, *AD* Alzheimer’s disease, *ALS* Amyotrophic lateral sclerosis

We used Cox proportional hazards models to evaluate the association of NfL and GFAP with incident neurodegenerative disease (Fig. 5). NfL and GFAP showed slight correlations with each other (*r* coefficients ranging from 0.14 to 0.22 across groups, *p* < 0.05), and thus, each plasma marker was tested in Cox models while adjusting for the other. As a first step, we assessed their association with a composite outcome defined by the occurrence of any of the neurodegenerative diagnoses of interest, with NfL showing a hazard ratio of 1.33 (95% CI, 1.28–1.39; *p* < 0.001) and GFAP a hazard ratio of 1.30 (95% CI, 1.24–1.37; *p* < 0.001). Because both NfL and GFAP were similarly associated with the risk of any neurodegenerative disease, we then evaluated the associations of NfL and GFAP with the risk of each neurodegenerative disease by conducting separate, diagnosis-specific analyses. For NfL, higher levels were associated with increased risk across all diseases even after full adjustment, with the strongest associations observed in ALS (HR 1.69; 95% CI, 1.58–1.80, *p* < 0.001), followed by APD (HR 1.51; 95% CI, 1.22–1.87, *p* < 0.001), AD (HR 1.31; 95% CI, 1.22–1.41, *p* < 0.001), and PD (HR 1.14; 95% CI, 1.05–1.23, *p* < 0.001). GFAP, by contrast, was a robust predictor for AD only (HR 1.72; 95% CI, 1.63–1.82, *p* < 0.001). No significant associations were found in PD or ALS, and only a trend toward increased risk was observed in APD (HR, 1.25; 95% CI, 0.89–1.76; *p* = 0.20).

The proportional hazards assumption was violated for NfL in AD and ALS and for GFAP in AD, indicating that the predictive effect of baseline biomarker levels changed over time. In AD, baseline NfL was strongly predictive in the short term but progressively attenuated, with HRs of 4.02 (95% CI, 3.08–5.24) at 2 years, 2.18 (95% CI, 1.90–2.50) at 5 years, and 1.37 (95% CI, 1.27–1.48) at 10 years. A similar pattern was observed for NfL in ALS, with HRs declining from 3.62 (95% CI, 3.23–4.04) at 2 years to 2.08 (95% CI, 1.91–2.27) at 5 years and 1.37 (95% CI, 1.23–1.54) at 10 years. GFAP in AD showed an even steeper early effect, with HRs of 13.3 (95% CI, 10.3–17.2) at 2 years, 4.64 (95% CI, 4.06–5.29) at 5 years, and 2.09 (95% CI, 1.96–2.23) at 10 years. By contrast, in PD and APD the proportional hazards assumption was met, indicating stable associations over time.

We also assessed the combined effect of NfL and GFAP (Supplementary Table 5). When dichotomized at the pathological threshold (*w*-score ≥1.5), NfL elevation alone was associated with increased risk across all diseases, particularly APD and ALS. In contrast, GFAP elevation alone was strongly associated with AD and, to a lesser extent, PD. Concomitant elevation of both biomarkers identified individuals at substantially higher risk compared with elevation of either biomarker alone, with HRs of 11.49 (95% CI, 8.44–15.64; *p* < 0.001) for AD, 7.83 (95% CI, 1.63–37.60; *p* = 0.01) for APD, 5.67 (95% CI, 2.75–11.67; *p* < 0.001) for ALS, and 1.95 (95% CI, 1.09–3.50; *p* = 0.02) for PD.

## Discussion

This study demonstrates distinct pre-diagnostic profiles of plasma NfL and GFAP protein levels across common neurodegenerative disorders, including ALS, AD, and parkinsonian syndromes. NfL was broadly associated with the risk of several neurodegenerative diseases, while GFAP was more selectively associated with AD.

In the era of target therapies aimed at slowing down the neurodegenerative process, it is crucial to identify patients at the earliest possible disease stage, when these treatments may be more effective [36–38]. Recent studies demonstrated that NfL and GFAP may be elevated years before patients manifest clinical symptoms of PD or AD. However, it is key to establish the specificity and practical context of using these biomarkers. For example, previous studies have highlighted that patients with high plasma NfL levels are at risk of developing PD [39, 40]. Still, we cannot overlook that some of these patients will develop other neurodegenerative diseases, such as ALS or AD, rather than PD, potentially impacting the inclusion of these patients into clinical trials. In this large population-based cohort study from the UK Biobank, we investigated independent and combined associations of plasma NfL and GFAP levels with future incident diagnoses of several major neurodegenerative diseases to shed light on the usefulness of these biomarkers in this challenging scenario.

Our findings revealed that NfL is a dynamic and sensitive marker of neuroaxonal injury, starting to increase several years before clinical diagnosis across all studied conditions. The degree of increase reflects the severity of neurodegeneration, showing the most pronounced rise in ALS, followed by APD, AD, and PD, which is a biologically plausible result. In contrast, GFAP displayed a more selective trajectory, with marked elevations primarily in individuals who later developed AD, while no significant associations were found with incident ALS or parkinsonian syndromes, suggesting astrocytic activation mainly in the pre-diagnostic phase of Alzheimer’s disease.

We also reconstructed trajectories of NfL and GFAP across the years preceding clinical diagnosis. A relevant question in this context is the time between biomarker elevation and disease onset. We found that NfL levels were associated with the time to diagnosis in most diseases. It had a straightforward rising trajectory in incident ALS cases only, with a steep progressive increase starting around 4 years before clinical diagnosis and a slight constant increase over time in the preclinical stages of other diseases. In addition, testing the proportional hazards assumption highlighted that the predictive value of NfL and GFAP was not constant over time. In AD and ALS, NfL showed strong short-term associations that progressively weakened with longer follow-up, with HR values >3 for the development of incident diseases within 2 years of follow-up. The same phenomenon was observed for GFAP in AD. These findings suggest especially high potential of these biomarkers for the identification of patients at risk of early appearance of neurodegenerative diseases within a few years, which may represent an ideal population for trials with neuroprotective treatments. In pre-diagnostic AD patients, both NfL and GFAP levels were significantly associated with incident disease; by comparing the two biomarkers, GFAP tended to increase even before NfL, with a larger percentage of patients showing elevated GFAP than NfL values; this data may reflect the distinct biological roles of the two biomarkers, suggesting that astrocytic activation astrogliosis might contribute to neuronal axonal damage in pre-diagnostic AD. Finally, we explored the relationship between NfL and GFAP levels in the pre-diagnostic phase of neurodegenerative diseases. Most previous studies have focused on either NfL or GFAP in isolation [17–20, 24], while the importance of their combined assessment and the elucidation of their complex interplay remained less explored. The combined presence of elevated NfL and GFAP *w*-scores (≥1.5) was consistently associated with higher HR than either biomarker alone, indicating that these proteins act independently but cumulatively to increase risk. Thus, the combined use of such biomarkers may be most helpful in identifying patients at risk of neurodegenerative diseases and be suitable for trials with nonspecific neuroprotective therapies against mechanisms shared across diseases (i.e., neuroinflammation or oxidative stress). Conversely, selecting patients for future therapies directed against specific molecular targets (i.e., amyloid, alpha-synuclein, etc.) may benefit from NfL and GFAP as screening tools but would likely require the addition of more disease-specific genetic, imaging, or fluid biomarkers.

This study has novelty and several strengths, including its comparative design across multiple neurodegenerative conditions in the same well-characterized population-based cohort, the standardized proteomic profiling, the careful inclusion/exclusion criteria and patient allocation procedures performed according to expert clinical reasoning, and the use of Cox models including many sociodemographic, lifestyle, clinical, and genetic factors, to account for the effect of many confounders, often overlooked in previous studies. Nonetheless, some limitations should also be acknowledged. First, the diagnoses were based on ICD-10 codes from health records, and some misdiagnoses might have occurred [41]. To minimize this risk, however, we selected only patients diagnosed by the NHS physicians rather than patient self-reports, and we excluded subjects with diagnostic uncertainty based on multiple ICD-10 codes and diseases potentially mimicking the neurodegenerative conditions of interest. Second, the atypical parkinsonism group was small, even after combining heterogeneous entities such as PSP and MSA, which may have limited statistical power and reduced the generalizability of results. Finally, this study is based on cross-sectional data, and future studies are warranted to explore longitudinal changes of the investigated biomarkers better.

In conclusion, this study reinforces the role of plasma NfL and GFAP in the pre-diagnostic phase of neurodegenerative disease, highlighting distinct profiles and trajectories across various disorders and suggesting that these biomarkers may provide information on which disease is the subject at risk for; moreover, their combined may help identify individuals at higher risk of incident neurodegeneration. These findings may be relevant to optimizing the selection strategies of target populations in the early disease stages to be included in future clinical trials with promising neuroprotective therapies.

## Supplementary Information

Below is the link to the electronic supplementary material.

Supplementary file1 (TIF 277 KB)

Supplementary file2 (DOCX 1035 KB)

## Data Availability

The UK Biobank is an open-access resource available at https://www.ukbiobank.ac.uk/researchers/. Data can be obtained from the UK Biobank by submitting a data request proposal. The data supporting this study’s findings were used under license for the current study (Application #147093) and are not publicly available.

## References

[1] Feigin VL, Vos T, Nichols E et al (2020) The global burden of neurological disorders: translating evidence into policy. Lancet Neurol 19(3):255–265. 10.1016/S1474-4422(19)30411-9 31813850 10.1016/S1474-4422(19)30411-9 PMC9945815

[2] Hansson O (2021) Biomarkers for neurodegenerative diseases. Nat Med 27(6):954–963. 10.1038/s41591-021-01382-x 34083813 10.1038/s41591-021-01382-x

[3] Jack CR Jr, Andrews JS, Beach TG et al (2024) Revised criteria for diagnosis and staging of Alzheimer’s disease: Alzheimer’s Association Workgroup. Alzheimers Dement 20(8):5143–5169. 10.1002/alz.13859 38934362 10.1002/alz.13859 PMC11350039

[4] Quattrone A, Franzmeier N, Levin J et al (2025) Prospective multicenter evaluation of the MDS “Suggestive of PSP” diagnostic criteria. Mov Disord 40(3):526–536. 10.1002/mds.30112 39797511 10.1002/mds.30112 PMC11926504

[5] Disanto G, Barro C, Benkert P et al (2017) Serum neurofilament light: a biomarker of neuronal damage in multiple sclerosis. Ann Neurol 81(6):857–870. 10.1002/ana.24954 28512753 10.1002/ana.24954 PMC5519945

[6] Gaetani L, Blennow K, Calabresi P, Di Filippo M, Parnetti L, Zetterberg H (2019) Neurofilament light chain as a biomarker in neurological disorders. J Neurol Neurosurg Psychiatry 90(8):870–881. 10.1136/jnnp-2018-320106 30967444 10.1136/jnnp-2018-320106

[7] Khalil M, Teunissen CE, Lehmann S et al (2024) Neurofilaments as biomarkers in neurological disorders - towards clinical application. Nat Rev Neurol 20(5):269–287. 10.1038/s41582-024-00955-x 38609644 10.1038/s41582-024-00955-x

[8] Ashton NJ, Janelidze S, Al Khleifat A et al (2021) A multicentre validation study of the diagnostic value of plasma neurofilament light. Nat Commun 12(1):3400. 10.1038/s41467-021-23620-z 34099648 10.1038/s41467-021-23620-z PMC8185001

[9] Hansson O, Janelidze S, Hall S et al (2017) Blood-based NfL: a biomarker for differential diagnosis of parkinsonian disorder. Neurology 7(10):930–937. 10.1212/WNL.0000000000003680 10.1212/WNL.0000000000003680 PMC5333515 28179466

[10] Delaby C, Alcolea D, Carmona-Iragui M et al (2020) Differential levels of neurofilament light protein in cerebrospinal fluid in patients with a wide range of neurodegenerative disorders. Sci Rep 10(1):9161. 10.1038/s41598-020-66090-x 32514050 10.1038/s41598-020-66090-x PMC7280194

[11] Bianco MG, Cristiani CM, Scaramuzzino L et al (2024) Combined blood neurofilament light chain and third ventricle width to differentiate progressive supranuclear palsy from Parkinson’s disease: a machine learning study. Parkinsonism Relat Disord 123:106978. 10.1016/j.parkreldis.2024.106978 38678852 10.1016/j.parkreldis.2024.106978

[12] Kou W, Li S, Yan R, Zhang J, Wan Z, Feng T (2025) Cerebrospinal fluid and blood neurofilament light chain in Parkinson’s disease and atypical parkinsonian syndromes: a systematic review and Bayesian network meta-analysis. J Neurol 272(4):311. 10.1007/s00415-025-13051-x 40180649 10.1007/s00415-025-13051-x

[13] Pedersen CC, Ushakova A, Alves G et al (2024) Serum neurofilament light at diagnosis: a prognostic indicator for accelerated disease progression in Parkinson’s disease. NPJ Parkinsons Dis 10:162. 10.1038/s41531-024-00768-1 39164268 10.1038/s41531-024-00768-1 PMC11336184

[14] Kuhle J, Kropshofer H, Haering DA et al (2019) Blood neurofilament light chain as a biomarker of MS disease activity and treatment response. Neurology 92(5):e1007–e1015. 10.1212/WNL.0000000000007032 30737333 10.1212/WNL.0000000000007032 PMC6442011

[15] Zetterberg H (2016) Neurofilament light: a dynamic cross-disease fluid biomarker for neurodegeneration. Neuron 6(91):1–3. 10.1016/j.neuron.2016.06.030 10.1016/j.neuron.2016.06.030 27387643

[16] Olsson B, Alberg L, Cullen NC et al (2019) NFL is a marker of treatment response in children with SMA treated with nusinersen. J Neurol 266(9):2129–2136. 10.1007/s00415-019-09389-8 31123861 10.1007/s00415-019-09389-8 PMC6687695

[17] Bjornevik K, O’Reilly EJ, Molsberry S et al (2021) Prediagnostic neurofilament light chain levels in amyotrophic lateral sclerosis. Neurology 11(15):e1466–e1474. 10.1212/WNL.0000000000012632 10.1212/WNL.0000000000012632 PMC8575132 34380747

[18] De Meyer S, Blujdea ER, Schaeverbeke JM et al (2025) Serum biomarkers as prognostic markers for Alzheimer’s disease in a clinical setting. Alzheimers Dement Diagn Assess Dis Monit 17(1):e70071. 10.1002/dad2.70071 10.1002/dad2.70071 PMC11780118 39886322

[19] You J, Wang L, Wang Y et al (2024) Prediction of future Parkinson disease using plasma proteins combined with clinical-demographic measures. Neurology 103:e209531. 10.1212/WNL.0000000000209531 38976826 10.1212/WNL.0000000000209531

[20] Preische O, Schultz SA, Apel A et al (2019) Serum neurofilament dynamics predicts neurodegeneration and clinical progression in presymptomatic Alzheimer’s disease. Nat Med 25(2):277–283. 10.1038/s41591-018-0304-3 30664784 10.1038/s41591-018-0304-3 PMC6367005

[21] Abdelhak A, Foschi M, Abu-Rumeileh S et al (2022) Blood GFAP as an emerging biomarker in brain and spinal cord disorders. Nat Rev Neurol 18(3):158–172. 10.1038/s41582-021-00616-3 35115728 10.1038/s41582-021-00616-3

[22] Gonzales MM, Wiedner C, Wang CP et al (2022) A population-based meta-analysis of circulating GFAP for cognition and dementia risk. Ann Clin Transl Neurol 9(10):1574–1585. 10.1002/acn3.51652 36056631 10.1002/acn3.51652 PMC9539381

[23] Peretti DE, Boccalini C, Ribaldi F et al (2024) Association of glial fibrillary acid protein, Alzheimer’s disease pathology and cognitive decline. Brain 147(3):4094–4104. 10.1093/brain/awae211 38940331 10.1093/brain/awae211 PMC11629700

[24] Pereira JB, Janelidze S, Smith R et al (2021) Plasma GFAP is an early marker of amyloid-β but not tau pathology in Alzheimer’s disease. Brain 16(11):3505–3516. 10.1093/brain/awab223 10.1093/brain/awab223 PMC8677538 34259835

[25] Arya R, Haque AKMA, Shakya H et al (2024) Parkinson’s disease: biomarkers for diagnosis and disease progression. Int J Mol Sci 25(22):12379. 10.3390/ijms252212379 39596444 10.3390/ijms252212379 PMC11594627

[26] Katzdobler S, Nübling G, Klietz M et al (2024) GFAP and NfL as fluid biomarkers for clinical disease severity and disease progression in multiple system atrophy (MSA). J Neurol 271(10):6991–6999. 10.1007/s00415-024-12647-z 39254698 10.1007/s00415-024-12647-z PMC11447157

[27] Huang SY, Chen SF, Cui M et al (2023) Plasma biomarkers and positron emission tomography tau pathology in progressive supranuclear palsy. Mov Disord 38(4):676–682. 10.1002/mds.29339 36781585 10.1002/mds.29339

[28] Heller C, Foiani MS, Moore K et al (2020) Plasma glial fibrillary acidic protein is raised in progranulin-associated frontotemporal dementia. J Neurol Neurosurg Psychiatry 91(3):263–270. 10.1136/jnnp-2019-321954 31937580 10.1136/jnnp-2019-321954

[29] Meier S, Willemse EAJ, Schaedelin S et al (2023) Serum glial fibrillary acidic protein compared with neurofilament light chain as a biomarker for disease progression in multiple sclerosis. JAMA Neurol 1:287–297. 10.1001/jamaneurol.2022.5250 10.1001/jamaneurol.2022.5250 PMC10011932 36745446

[30] Bentivenga GM, Gonzalez-Ortiz F, Baiardi S et al (2025) Clinical value of novel blood-based tau biomarkers in Creutzfeldt-Jakob disease. Alzheimers Dement 21(2):e14422. 10.1002/alz.14422 39641397 10.1002/alz.14422 PMC11848332

[31] Oeckl P, Weydt P, Steinacker P et al (2019) Different neuroinflammatory profile in amyotrophic lateral sclerosis and frontotemporal dementia is linked to the clinical phase. J Neurol Neurosurg Psychiatry 90(1):4–10. 10.1136/jnnp-2018-318868 30224549 10.1136/jnnp-2018-318868

[32] Benedet AL, Milà-Alomà M, Vrillon A et al (2021) Differences between plasma and cerebrospinal fluid glial fibrillary acidic protein levels across the Alzheimer disease continuum. JAMA Neurol 1:1471–1483. 10.1001/jamaneurol.2021.3671 10.1001/jamaneurol.2021.3671 PMC8524356 34661615

[33] Wang X, Shi Z, Qiu Y, Sun D, Zhou H (2024) Peripheral GFAP and NfL as early biomarkers for dementia: longitudinal insights from the UK Biobank. BMC Med 22(1):192. 10.1186/s12916-024-03418-8 38735950 10.1186/s12916-024-03418-8 PMC11089788

[34] Freedman MS, Gnanapavan S, Booth RA et al (2024) Guidance for use of neurofilament light chain as a cerebrospinal fluid and blood biomarker in multiple sclerosis management. EBioMedicine 101:104970. 10.1016/j.ebiom.2024.104970 38354532 10.1016/j.ebiom.2024.104970 PMC10875256

[35] Delaby C, Ladang A, Martinez-Yriarte J et al (2025) Clinical use and reporting of neurofilament quantification in neurological disorders: a global overview. Alzheimers Dement 21(6):e70343. 10.1002/alz.70343 40551293 10.1002/alz.70343 PMC12185249

[36] Quattrone A, Franzmeier N, Huppertz HJ et al (2024) Magnetic resonance imaging measures to track atrophy progression in progressive supranuclear palsy in clinical trials. Mov Disord 39(8):1329–1342. 10.1002/mds.29866 38825840 10.1002/mds.29866

[37] Sims JR, Zimmer JA, Evans CD et al (2023) Donanemab in early symptomatic Alzheimer disease: the TRAILBLAZER-ALZ 2 randomized clinical trial. JAMA 330(8):512–527. 10.1001/jama.2023.13239 37459141 10.1001/jama.2023.13239 PMC10352931

[38] Abate F, Di Biasio F, Marchese R et al (2025) Clinical trial eligibility in PSP: population representativeness and potential criteria adjustment based on PSP-NET findings. Parkinsonism Relat Disord 131:107226. 10.1016/j.parkreldis.2024.107226 39700727 10.1016/j.parkreldis.2024.107226

[39] Pilotto A, Ashton NJ, Lupini A et al (2024) Plasma NfL, GFAP, amyloid, and p-tau species as prognostic biomarkers in Parkinson’s disease. J Neurol 271(12):7537–7546. 10.1007/s00415-024-12669-7 39249107 10.1007/s00415-024-12669-7 PMC11588809

[40] Liguori C, Fernandes M, Zatti C et al (2025) Plasma NfL, GFAP and pTau181 in patients with isolated REM sleep behaviour disorder. Sleep. 10.1093/sleep/zsaf163 40488440 10.1093/sleep/zsaf163

[41] Lo RY (2025) To trust or not to trust? ICD-coded diagnosis of Parkinson’s disease. Mov Disord 40(2):181–183. 10.1002/mds.30105 39962630 10.1002/mds.30105

