# Supplementary figures and images for: Plasma NfL and GFAP in the preclinical stages of neurodegenerative diseases: insights from the UK Biobank
Source: J Neurol. 2025 Nov 9;272(12):755. doi: 10.1007/s00415-025-13498-y (PMC12597848; doi:10.1007/s00415-025-13498-y)

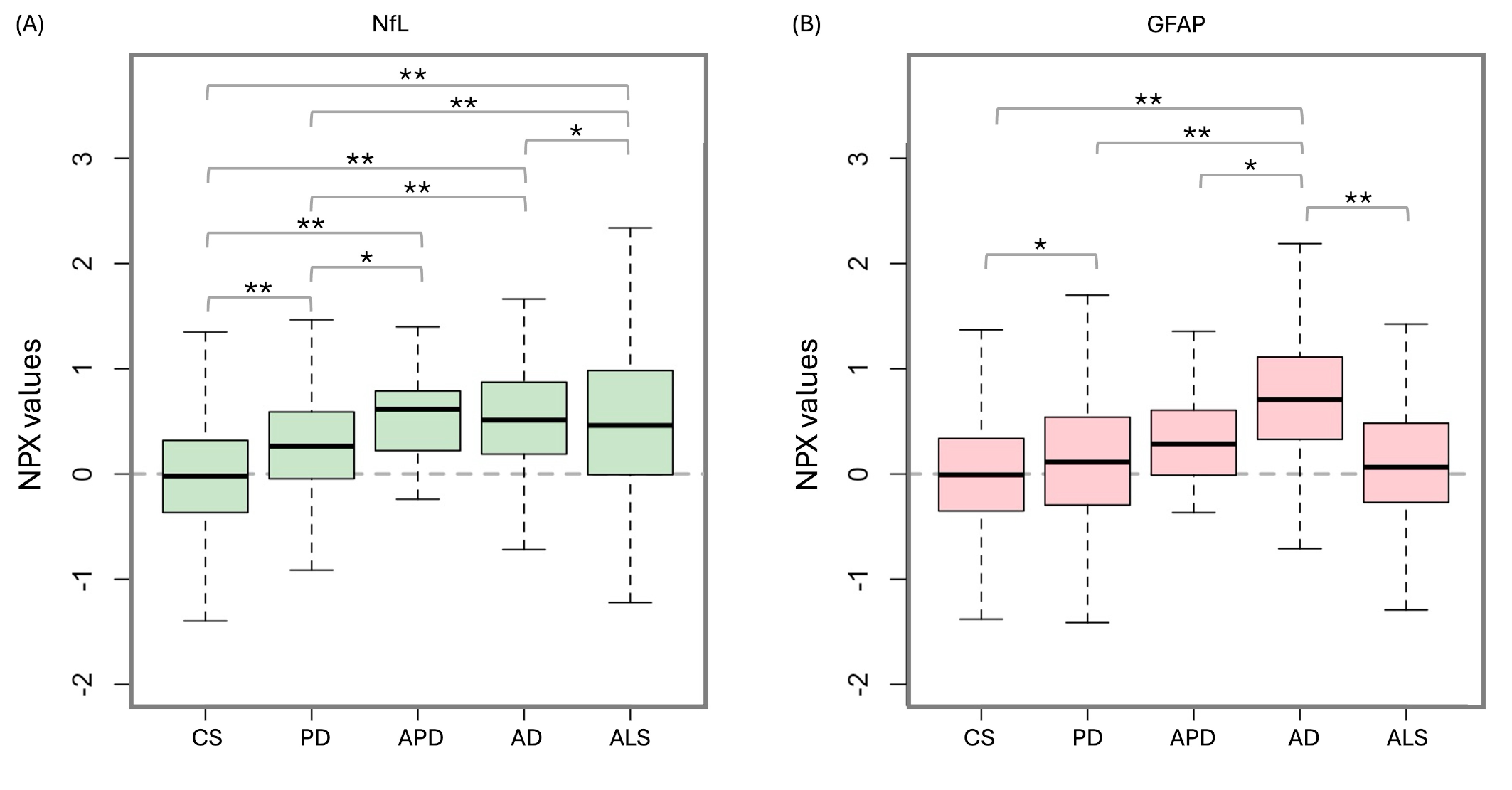

Supplement: Supplementary file 1 — Supplementary file1 (TIF 277 KB) [file 415_2025_13498_MOESM1_ESM.tif]
