# Supplementary material for: Plasma NfL and GFAP in the preclinical stages of neurodegenerative diseases: insights from the UK Biobank
Source: J Neurol. 2025 Nov 9;272(12):755. doi: 10.1007/s00415-025-13498-y (PMC12597848; doi:10.1007/s00415-025-13498-y)
Supplement: Supplementary file 2 — Supplementary file2 (DOCX 1035 KB) [file 415_2025_13498_MOESM2_ESM.docx]

**Supplementary Materials**

*1. Observation periods and time definitions*

Several time metrics were defined to characterize the observation period and perform time-to-event analyses. These definitions ensured consistency across analyses and allowed accurate estimation of follow-up times:

- Baseline date: defined as the date participants attended the assessment centre for their initial visit (UK Biobank Field ID: 53, "Date of attending assessment centre"). This date corresponds to the blood sample collection used to measure neurofilament light chain (NfL) and glial fibrillary acidic protein (GFAP) levels.
- Date of death: retrieved from linked national mortality registries provided by UK Biobank (Field ID: 40000, "Date of death"). This date was used to define censoring and time-to-event in survival analyses.
- Date of loss to follow-up: participants were considered lost to follow-up if their primary care or hospital records were incomplete or if they withdrew consent during the observation period. This information was obtained from the withdrawal status and administrative health record linkage (Field ID: 191, "Date lost to follow-up").
- Date of diagnosis: for each neurodegenerative disease of interest, the date of diagnosis was defined as the earliest recorded ICD-10 code corresponding to the specific condition, obtained from hospital inpatient records, primary care data, or death registries. Specifically:
  - Parkinson's disease (PD): Field ID 131022
  - Atypical Parkinsonian Disorders (APD): PSP Field ID: 42034; MSA Field ID: 42036. For comprehensive classification, PD Field ID 131022 was also considered. The date of diagnosis was defined in incident PK cases as the earliest recorded date among any of these Field IDs.
  - Alzheimer's disease (AD): Primary Field ID: 42020. To ensure comprehensive classification, additional dementia-related ICD-10 codes were included: Field IDs 130836, 131036, 130840, 130842. The date of diagnosis in incident AD cases was defined as the earliest recorded date among any of these Field IDs.
  - Amyotrophic lateral sclerosis (ALS): Field ID 131016
- Time to dementia in PD: defined as the interval between the date of Parkinson’s disease diagnosis (Field ID: 131022) and the first occurrence of any dementia-related ICD-10 code (Field IDs: 130836, 131036, 131038, 42024, 130840, 130842).
- Censoring date: the administrative censoring date was set to November 30, 2022, corresponding to the latest date up to which mortality data had been uploaded and made available by the UK Biobank at the time the datasets were downloaded (February 2025). This date was used as the end of follow-up for participants who had not received a diagnosis of interest and had neither died nor been lost to follow-up before that point.

Based on these definitions, three distinct time metrics were calculated:

- Total observation time: defined as the time elapsed between the baseline assessment date and the earliest occurrence of death, loss to follow-up, or censoring date. This measure reflects the overall observation period in the cohort and was reported descriptively in the main text.
- Time-to-event for Cox analyses: for the Cox proportional hazards models, time-to-event was defined as the time between baseline and the earliest occurrence of the diagnosis of interest, death, or censoring date. In models focused on a specific diagnosis (e.g., Parkinson’s disease), participants who developed a different neurodegenerative condition (e.g., AD, ALS, or APD) were censored at the date of that alternative diagnosis, death, or censoring, whichever came first. Control subjects were censored at the earliest of death or the censoring date, if no event occurred.
- Time to diagnosis: for incident cases (participants who developed PD, PSP, MSA, AD or ALS during the observation period), time to diagnosis was calculated as the interval between the baseline assessment and the earliest date of diagnosis.

*2. Health-related outcomes*

Incident diagnoses of major neurodegenerative diseases were identified in the UK Biobank using ICD-10 codes. PD was defined by ICD-10 as G20, PSP as G23.1, MSA as G23.2, G23.3 or G90.3, AD as G30 or F00, and ALS as G12.2. PSP and MSA cases were considered together as “atypical parkinsonian disorders” group. For participants with multiple ICD-10 diagnoses of interest (AD, PD, APD, ALS), disease group allocation followed the hierarchy outlined in Supplementary Table 2. Participants with ICD-10-based diagnosis of both PD and APD (either PSP or MSA) were classified as PK, reflecting the frequent misclassification of atypical parkinsonism as PD in the early stages. For this reason, in these patients, the disease onset was defined as the earliest date of either an ICD-10 diagnosis of PSP, MSA or PD. Motor neurodegenerative disorders (PD, APD, ALS) were prioritized over cognitive disorders (AD), based on the rationale that some cognitive impairment is frequently observed in motor syndromes, whereas prominent motor features are rare in Alzheimer’s disease. Participants with concurrent diagnoses of PD and ALS (n = 8), or ALS and PSP (n = 21), were excluded from both groups due to diagnostic uncertainty, as it was not possible to determine which condition represented the primary clinical picture. Other exclusion procedures were performed according to the following steps: first, subjects with ICD-10 codes of inflammatory, infectious, acute cerebrovascular diseases, head trauma, or demyelinating neurological disorders potentially influencing NfL or GFAP levels were excluded from all patient and control groups. Second, subjects with ICD-10 codes of specific disorders potentially mimicking the diseases of interest were excluded from patient groups based on clinical reasoning to increase diagnostic specificity (i.e. excluding “secondary parkinsonism” from PD, or “fronto-temporal dementia” from the AD group). The full list of excluded conditions is provided in Supplementary Table 1. Since PD is a common disease and G20 is likely a widely used ICD-10 code, the PD group underwent a further refinement. For participants diagnosed with both PD and dementia, the time to dementia was calculated as the interval between the date of PD diagnosis and the date of dementia diagnosis. The date of dementia diagnosis was determined as the earliest recorded diagnosis among multiple dementia-related ICD-10 codes, ensuring a comprehensive classification. Participants with a time to dementia from PD diagnosis of less than two years were excluded, as they were more likely to have alternative neurodegenerative conditions, such as dementia with Lewy bodies or other dementia-parkinsonism syndromes. Control subjects (CS) were participants with no neurodegenerative diseases of interest and no confounding or mimicking neurological conditions (Supplementary Table 1). Within this group, a subset of healthy controls (HC) was defined as individuals with no ICD-10 diagnoses at all (neurologic or non-neurologic diseases) for w-score calculation of the variables of interest.

*3. W-score calculation*

W-scores are standardized scores, conceptually like z-scores, but adjusted for relevant demographic and anthropometric covariates that may influence the variables of interest (age, sex, and body mass index [BMI] when applied to NfL levels and hand grip strength; age and sex for GFAP levels; and age, sex, and years of education for cognitive performance measures). To calculate w-scores, we used a reference population of 4,926 healthy controls (HC), defined as participants without ICD-10 diagnoses and without loss to follow-up. Linear regression models were fitted in this group with the variable of interest (i.e., NfL or GFAP) as the dependent variable and relevant covariates as independent variables. The models were then applied to the cohort to calculate residuals of the variables of interest, subtracting the contribution of covariates. Finally, w-scores were calculated as z-scores of the residuals of variables of interest. Exactly like z-scores, the resulting w-scores reflect how much an individual’s observed value deviates from the expected value, normalized to the distribution in the healthy population. Positive w-scores indicate values higher than expected, whereas negative w-scores indicate values lower than expected.

*4. Variable Derivation Procedures*

- eGFR: the eGFR was calculated for each participant using the 2021 Chronic Kidney Disease Epidemiology Collaboration (CKD-EPI) formula, which incorporates both serum creatinine and cystatin C levels to provide a more accurate estimation of kidney function [1]. The formula applied was:


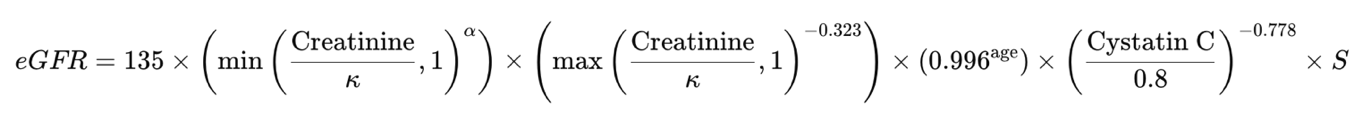


where κ and α were sex-specific constants (κ = 0.7 and α = −0.241 for females; κ = 0.9 and α = −0.302 for males), and S was set to 1.012 for females and 1.000 for males. Age referred to the age at recruitment, and serum creatinine and cystatin C levels were expressed in mg/dL and obtained at baseline (Instance 0).

- Years of education: years of education were estimated based on participants’ reported educational qualifications using the International Standard Classification of Education (ISCED) framework (https://uis.unesco.org/sites/default/files/documents/international-standard-classification-of-education-isced-2011-en.pdf). Specifically, participants were assigned 7 years if reporting no qualifications, 10 years for a Certificate of Secondary Education (CSE) or equivalent, 11 years for O-levels/GCSEs, 13 years for A-levels or equivalent, and 15 years for a college or university degree. Participants who answered "Prefer not to answer" or "Do not know" were coded as missing for this variable. Years of education were used as an adjustment variable in cognitive performance analyses.
- Family history: family history for PD and AD/dementia was determined based on participant-reported information on illnesses diagnosed in first-degree relatives, specifically in the father (Field ID: 20107), mother (Field ID: 20110), and siblings (Field ID: 20111). A family history of PD was assigned when this disease was reported in at least one of these relatives. Similarly, a family history of AD/dementia was defined when either Alzheimer’s disease or dementia was reported in at least one first-degree relative. Binary variables were generated to indicate the presence or absence of familiarity for each condition.

*5. Cox Proportional Hazards Models*

Cox proportional hazards models were used to assess the association between baseline plasma levels of NfL and GFAP and the risk of developing neurodegenerative diseases (PD, APD, AD and ALS). A stepwise adjustment strategy was employed across six sequential models, applied uniformly across all disease groups:

- Model 1 (unadjusted): included only the biomarkers of interest (NfL and GFAP).
- Model 2 (sociodemographic factors): further included Townsend deprivation index, ethnicity, and educational qualifications.
- Model 3 (lifestyle factors): added smoking status and alcohol consumption.
- Model 4 (clinical variables): included diabetes, hypertension, and estimated glomerular filtration rate (eGFR category).
- Model 5 (genetic risk): incorporated APOE-ε4 status, polygenic risk scores (PRS) for PD and AD, and family history of PD and AD/dementia in first-degree relatives.
- Model 6 (fully adjusted): included all covariates listed above.

*6.* ***Cox Regression Stratified by Combined Biomarker Elevation (w-score ≥ 1.5)***

Participants were stratified into four groups based on their plasma NfL and GFAP levels, using a w-score threshold of 1.5 to define elevated concentrations. The reference group included individuals with both biomarkers below the 1.5 w-score threshold. The other three groups included participants with: (i) elevated NfL (≥1.5) and GFAP below threshold, (ii) elevated GFAP (≥1.5) and NfL below threshold, and (iii) both biomarkers elevated (≥1.5). For each neurodegenerative disease of interest (Parkinson’s disease, atypical parkinsonism, Alzheimer’s disease, and amyotrophic lateral sclerosis), separate Cox proportional hazards models were fitted. Each group with at least one biomarker elevated was compared individually to the reference group (both biomarkers below threshold), resulting in three distinct comparisons per model: elevated NfL vs. reference, elevated GFAP vs. reference, both elevated vs. reference. The definition of time-to-event and the covariates included in the six adjustment models followed the same approach described previously.

**Supplementary Table 1.** Confounding neurological conditions or mimicking diseases excluded from participant groups based on ICD-10 codes.

| **ICD-10 code** | **Diagnosis** | **Excluded from** |
| --- | --- | --- |
| *ICD-10 codes of confounding neurological disorders* | | |
| G00-09 | Inflammatory diseases of the central nervous system | CS, PD, APD, AD, ALS |
| G35 | Multiple sclerosis | CS, PD, APD, AD, ALS |
| G36 | Other acute disseminated demyelination | CS, PD, APD, AD, ALS |
| G37 | Other demyelinating diseases of CNS | CS, PD, APD, AD, ALS |
| I60-I66 | Acute cerebrovascular diseases* | CS, PD, APD, AD, ALS |
| S02, S06, S07, S09 | Traumatic Brain injury | CS, PD, APD, AD, ALS |
| *ICD-10 codes of disorders mimicking diseases of interest*** | | |
| G21 | Secondary Parkinsonism | CS, PD |
| G22 | Parkinsonism in diseases classified elsewhere | CS, PD |
| G23*** | Other degenerative diseases of basal ganglia | CS, PD |
| F01 | Vascular dementia | CS, AD |
| G31.0, F02.0 | Frontotemporal dementia | CS, AD |
| G12.0, G12.1, G12.8, G12.9 | Inherited and unspecified spinal muscular atrophies | CS, ALS |
| G70-73 | Neuromuscular disorders | CS, ALS |
| M33 | Dermatopolymyositis | CS, ALS |
| *General ICD-10 codes* | | |
| F02 | Dementia in other diseases classified elsewhere | CS |
| F03 | Unspecified dementia | CS |
| F04 | Organic amnesic syndrome, not induced by alcohol/drugs | CS |

*Abbreviations: CS = control subjects; PD = Parkinson's disease; APD = atypical parkinsonian disorders; AD = Alzheimer's disease; ALS = amyotrophic lateral sclerosis.*

Exclusion procedures were performed according to three steps:

1. Subjects with ICD-10 codes of inflammatory, infectious, acute cerebrovascular diseases, head trauma, or demyelinating neurological disorders potentially influencing NfL or GFAP levels were excluded from all patient and control groups.
2. Subjects with ICD codes of specific disorders potentially mimicking the diseases of interest were excluded from patient groups based on clinical reasoning to increase diagnostic specificity (i.e. excluding “secondary parkinsonism” and “other degenerative diseases of basal ganglia” from PD, or “vascular dementia” and “fronto-temporal dementia” from Alzheimer’s disease), to reduce potential misdiagnoses.
3. Subject with ICD codes of general terms such as “unspecified dementia” or “organic amnesic syndrome” were excluded from controls but not from patients since these terms may be used to describe symptoms and these ICD codes were commonly coupled with codes of more specific diseases.

*Acute cerebrovascular disease cases were defined as participants with a recorded diagnosis of subarachnoid hemorrhage, intracerebral hemorrhage, cerebral infarction, or other nontraumatic intracranial hemorrhages (ICD-10 codes: I60–I66) occurring before baseline assessment (Instance 0).

**The group of participants with incident PD was further refined by excluding subjects with a diagnosis of any dementia (G30, G31, F01, F02, F03) within 2 years of the PD diagnosis, as they were considered more likely to have alternative neurodegenerative conditions, such as dementia with Lewy bodies or other dementia-parkinsonism syndromes.

***G23 includes codes of atypical parkinsonism (progressive supranuclear palsy and multiple system atrophy).

**Supplementary Table 2.** Diagnostic hierarchy used to assign participants with multiple ICD-10 diagnoses of interest to a single primary disease group.

|  | **PD** | **APD** | **AD** | **ALS** |
| --- | --- | --- | --- | --- |
| **PD** | PD | - | - | - |
| **APD** | APD | APD | - | - |
| **AD** | PD* | APD | AD | - |
| **ALS** | excluded | excluded | ALS | ALS |

*Abbreviations: CS = control subjects; PD = Parkinson's disease; APD = atypical parkinsonian disorders; AD = Alzheimer's disease; ALS = amyotrophic lateral sclerosis.*

When individuals had more than one ICD-10 diagnosis among PD, APD (including progressive supranuclear palsy [PSP] and multiple system atrophy [MSA]), AD, and ALS, group allocation followed a predefined hierarchy prioritizing motor neurodegenerative disorders (PD, APD, ALS) over cognitive disorders (AD), reflecting the clinical observation that cognitive symptoms are commonly seen in motor syndromes, while motor symptoms are rarely prominent in Alzheimer’s disease, and when present, they may suggest alternative diagnoses. Participants with ICD-10-based diagnosis of both PD and APD (either PSP or MSA) were classified as APD, reflecting the frequent misclassification of atypical parkinsonism as PD in the early stages. Participants with concurrent diagnoses of PD and ALS (n = 8), or ALS and PSP (n = 21), were excluded from both groups due to diagnostic uncertainty, as it was not possible to determine which condition represented the primary clinical picture.

*After group assignment, participants with both PD and a dementia diagnosis were retained in the PD group only if dementia occurred more than two years after the PD diagnosis. Those with earlier onset of dementia were excluded, as early cognitive decline may indicate alternative conditions such as dementia with Lewy bodies or other dementia-parkinsonism syndromes.

| **Variable category** | **Variable description** | **UK Biobank Field ID(s)** | **Notes** |
| --- | --- | --- | --- |
| *Demographics* | Age at recruitment | 21022 |  |
|  | Sex | 31 |  |
| *Anthropometrics* | Body Mass Index | 21001 | at baseline (Instance 0) |
| *Cognitive performances* | Fluid intelligence score | 20016 | at baseline (Instance 0) |
|  | Reaction time (time to correctly identify matches) | 20023 | at baseline (Instance 0) |
| *Physical performance* | Hand grip strength (mean) | 46, 47 | at baseline (Instance 0); mean of right- and left-hand measurements |
| *Socioeconomic variables* | Townsend deprivation index | 22189 |  |
|  | Ethnicity | 21000 |  |
|  | Educational qualifications | 6138 |  |
|  | Years of education | derived variable | calculated from educational qualifications |
| *Lifestyle factors* | Smoking status | 20116 |  |
|  | Alcohol consumption | 20117 |  |
| *Comorbidities* | Diabetes | 41270 | ICD-10 codes: E10, E11, E12, E13, E14, O24.0, O24.1, O24.3 |
|  | Hypertension | 41270 | ICD-10 codes: I10, I11, I12, I13, I15 |
|  | Kidney function (eGFR) | 30700, 30720 | based on serum cystatin C and creatinine |
| *Genetic predisposition* | Family history of PD | 20107, 20110, 20111 | diagnosis in first-degree relatives (parents, siblings) |
|  | Family history of AD or dementia | 20107, 20110, 20111 | diagnosis in first-degree relatives (parents, siblings) |
|  | PRS for PD | 26260 | calculated based on genome-wide data |
|  | PRS for AD | 26206 | calculated based on genome-wide data |
|  | Apolipoprotein E genotype | 22182, 22185 | based on rs429358 and rs7412 variants |

**Supplementary Table 3.** Variables included in the study and their corresponding UK Biobank Field IDs.

*Abbreviations: eGFR = estimated glomerular filtration rate; PD = Parkinson’s disease; AD = Alzheimer’s disease.*

**Supplementary Table 4.** Availability of apolipoprotein E genotype data, polygenic risk scores, and educational qualifications across study groups.

|  | **CS**  **(n = 44,107)** | **PD**  **(n = 505)** | **APD**  **(n = 26)** | **AD**  **(n =476)** | **ALS**  **(n = 189)** |
| --- | --- | --- | --- | --- | --- |
| *APOE* | 42,229 | 486 | 25 | 453 | 182 |
| *PRS-PD* | 43,645 | 500 | 26 | 470 | 187 |
| *PRS-AD* | 43,645 | 500 | 26 | 470 | 187 |
| *Educational qualifications* | 41,061 | 462 | 24 | 438 | 176 |
| *Fluid intelligence score* | 13,258 | 147 | 9 | 106 | 55 |
| *Reaction time* | 40,678 | 458 | 23 | 425 | 174 |
| *Hand grip* | 41,357 | 463 | 23 | 453 | 179 |

*Abbreviations: APOE = apolipoprotein E; CS = control subjects; PD = Parkinson’s disease; APD = atypical parkinsonian disorders; AD = Alzheimer’s disease; ALS = amyotrophic lateral sclerosis; PRS = polygenic risk score.*

**Supplementary Table 5.** Hazard ratios of elevated NfL and GFAP levels for incident neurodegenerative diseases.

|  |  | NfL w-score ≥ 1.5 | | | GFAP w-score ≥ 1.5 | | | Both NfL and GFAP w-score ≥ 1.5 | | | |
| --- | --- | --- | --- | --- | --- | --- | --- | --- | --- | --- | --- |
|  |  | HR | CI 95% | p value | HR | CI 95% | p value | HR | CI 95% | p value | |
| PD | Model 1 | 1.68 | 1.25-2.27 | **<0.001** | 2.00 | 1.48-2.48 | **<0.001** | 2.37 | 1.34-4.22 | | **0.003** |
|  | Model 2 | 1.68 | 1.25-2.26 | **<0.001** | 2.00 | 1.47-2.71 | **<0.001** | 2.37 | 1.33-4.21 | | **0.003** |
|  | Model 3 | 1.69 | 1.25-2.28 | **<0.001** | 1.95 | 1.44-2.64 | **<0.001** | 2.29 | 1.29-4.07 | | **0.005** |
|  | Model 4 | 1.46 | 1.08-1.98 | **0.02** | 1.95 | 1.44-2.65 | **<0.001** | 2.06 | 1.15-3.71 | | **0.02** |
|  | Model 5 | 1.67 | 1.24-2.25 | **<0.001** | 1.99 | 1.46-2.69 | **<0.001** | 2.30 | 1.29-4.09 | | **0.005** |
|  | Model 6 | 1.46 | 1.07-1.98 | **0.02** | 1.90 | 1.40-2.58 | **<0.001** | 1.95 | 1.09-3.50 | | **0.02** |
| APD | Model 1 | 6.25 | 2.53-15.52 | **<0.001** | 1.11 | 0.15-8.41 | 0.92 | 10.52 | 2.39-46.32 | | **0.002** |
|  | Model 2 | 6.28 | 2.53-15.58 | **<0.001** | 1.08 | 0.14-8.25 | 0.94 | 9.98 | 2.27-43.95 | | **0.002** |
|  | Model 3 | 6.33 | 2.55-15.70 | **<0.001** | 1.13 | 0.15-8.58 | 0.91 | 10.88 | 2.47-47.99 | | **0.002** |
|  | Model 4 | 5.04 | 1.95-13.02 | **<0.001** | 1.07 | 0.14-8.17 | 0.95 | 7.82 | 1.65-36.95 | | **0.009** |
|  | Model 5 | 6.36 | 2.57-15.77 | **<0.001** | 1.13 | 0.15-8.59 | 0.91 | 10.78 | 2.45-47.52 | | **0.002** |
|  | Model 6 | 5.34 | 2.07-13.76 | **<0.001** | 1.09 | 0.14-8.31 | 0.93 | 7.83 | 1.63-37.60 | | **0.01** |
| AD | Model 1 | 2.30 | 1.69-3.14 | **<0.001** | 5.89 | 4.67-7.43 | **<0.001** | 15.27 | 11.33-20.56 | | **<0.001** |
|  | Model 2 | 2.29 | 1.68-3.12 | **<0.001** | 5.84 | 4.63-7.37 | **<0.001** | 14.87 | 11.03-20.03 | | **<0.001** |
|  | Model 3 | 2.27 | 1.66-3.10 | **<0.001** | 5.74 | 4.55-7.25 | **<0.001** | 14.64 | 10.86-19.73 | | **<0.001** |
|  | Model 4 | 2.06 | 1.51-2.83 | **<0.001** | 5.79 | 4.59-7.31 | **<0.001** | 14.38 | 10.59-19.53 | | **<0.001** |
|  | Model 5 | 2.22 | 1.63-3.03 | **<0.001** | 5.01 | 3.97-6.33 | **<0.001** | 12.94 | 9.60-17.45 | | **<0.001** |
|  | Model 6 | 1.97 | 1.44-2.70 | **<0.001** | 4.66 | 3.69-5.89 | **<0.001** | 11.49 | 8.44-15.64 | | **<0.001** |
| ALS | Model 1 | 5.63 | 4.03-7.86 | **<0.001** | 1.67 | 0.92-3.04 | 0.09 | 5.17 | 2.52-10.60 | | **<0.001** |
|  | Model 2 | 5.58 | 4.00-7.80 | **<0.001** | 1.66 | 0.92-3.02 | 0.09 | 5.11 | 2.49-10.48 | | **<0.001** |
|  | Model 3 | 5.62 | 4.02-7.85 | **<0.001** | 1.68 | 0.93-3.05 | 0.09 | 5.21 | 2.54-10.69 | | **<0.001** |
|  | Model 4 | 5.78 | 4.12-8.10 | **<0.001** | 1.67 | 0.92-3.02 | 0.09 | 5.75 | 2.75-11.63 | | **<0.001** |
|  | Model 5 | 5.62 | 4.02-7.85 | **<0.001** | 1.67 | 0.92-3.04 | 0.09 | 5.18 | 2.53-10.62 | | **<0.001** |
|  | Model 6 | 5.73 | 4.09-8.04 | **<0.001** | 1.67 | 0.92-3.03 | 0.09 | 5.67 | 2.75-11.67 | | **<0.001** |

NfL and GFAP w-scores were binarized as elevated or normal, as defined above (w-score ≥1.5), and Cox models were repeated using these variables rather than continuous w-score values. Cox proportional hazards models were used to estimate hazard ratios (HR), 95% confidence intervals (CI), and p-values for each group compared to control subjects. Six models were applied, adjusting for potential confounders (see Supplementary Materials for full model specification). *Abbreviations: HR = hazard ratio; CI = confidence interval; NfL = neurofilament light chain; GFAP = glial fibrillary acidic protein; PD = Parkinson’s disease; APD = atypical parkinsonian disorders; AD = Alzheimer’s disease; ALS = amyotrophic lateral sclerosis.*

**Supplementary Figure 1.** Distribution of NfL (A) and GFAP (B) NPX values across groups.


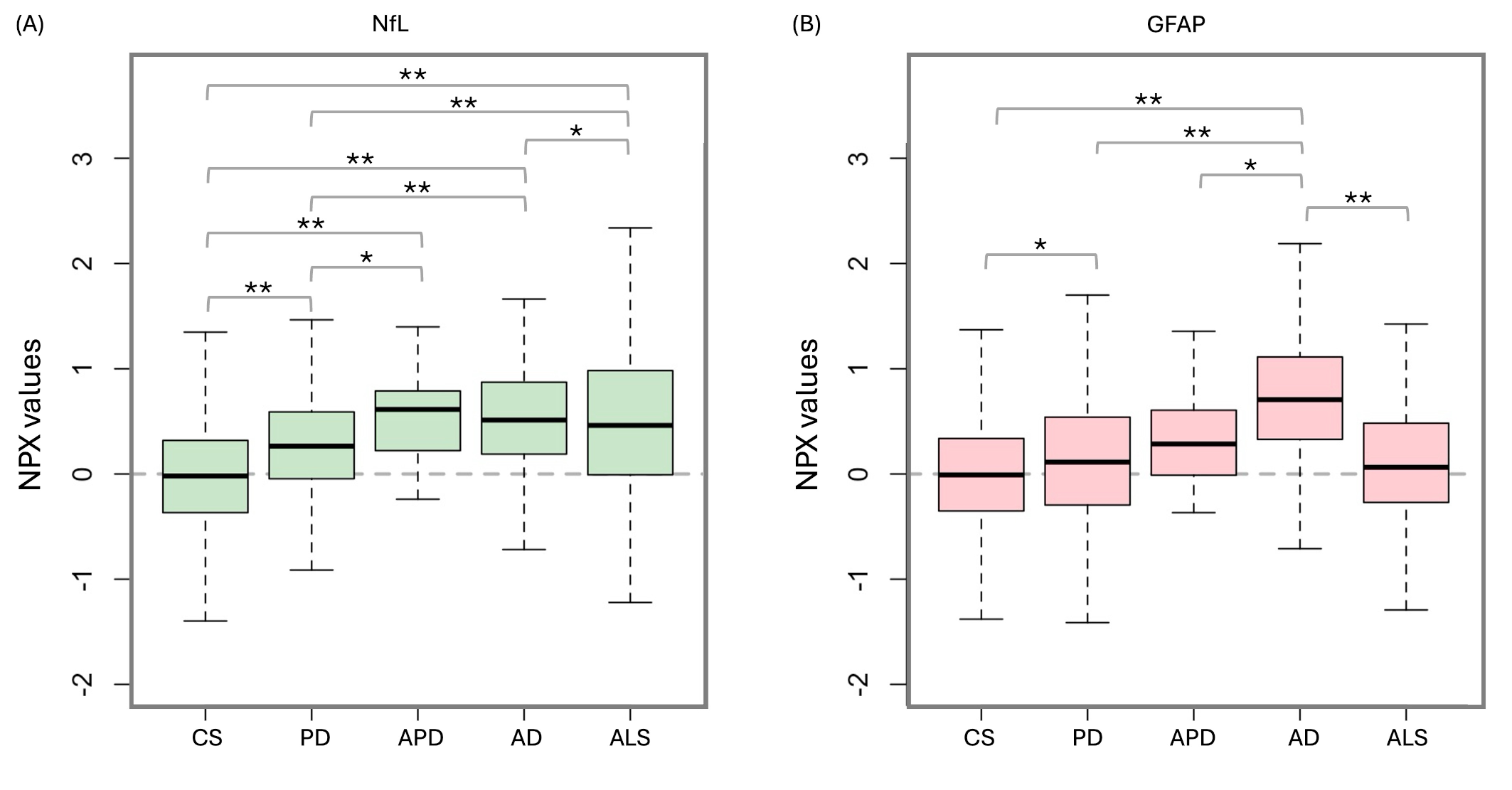


Pairwise comparisons were performed on raw biomarker values with estimated glomerular filtration rate as covariate. Horizontal brackets indicate statistically significant differences from post hoc comparisons (*p < 0.05; **p < 0.001). Outliers are not shown in the plots for visual representation. *Abbreviations: CS = control subjects; PD = Parkinson’s disease; APD = atypical parkinsonian disorders; AD = Alzheimer’s disease; ALS = amyotrophic lateral sclerosis; NfL = neurofilament light chain; GFAP = glial fibrillary acidic protein.*


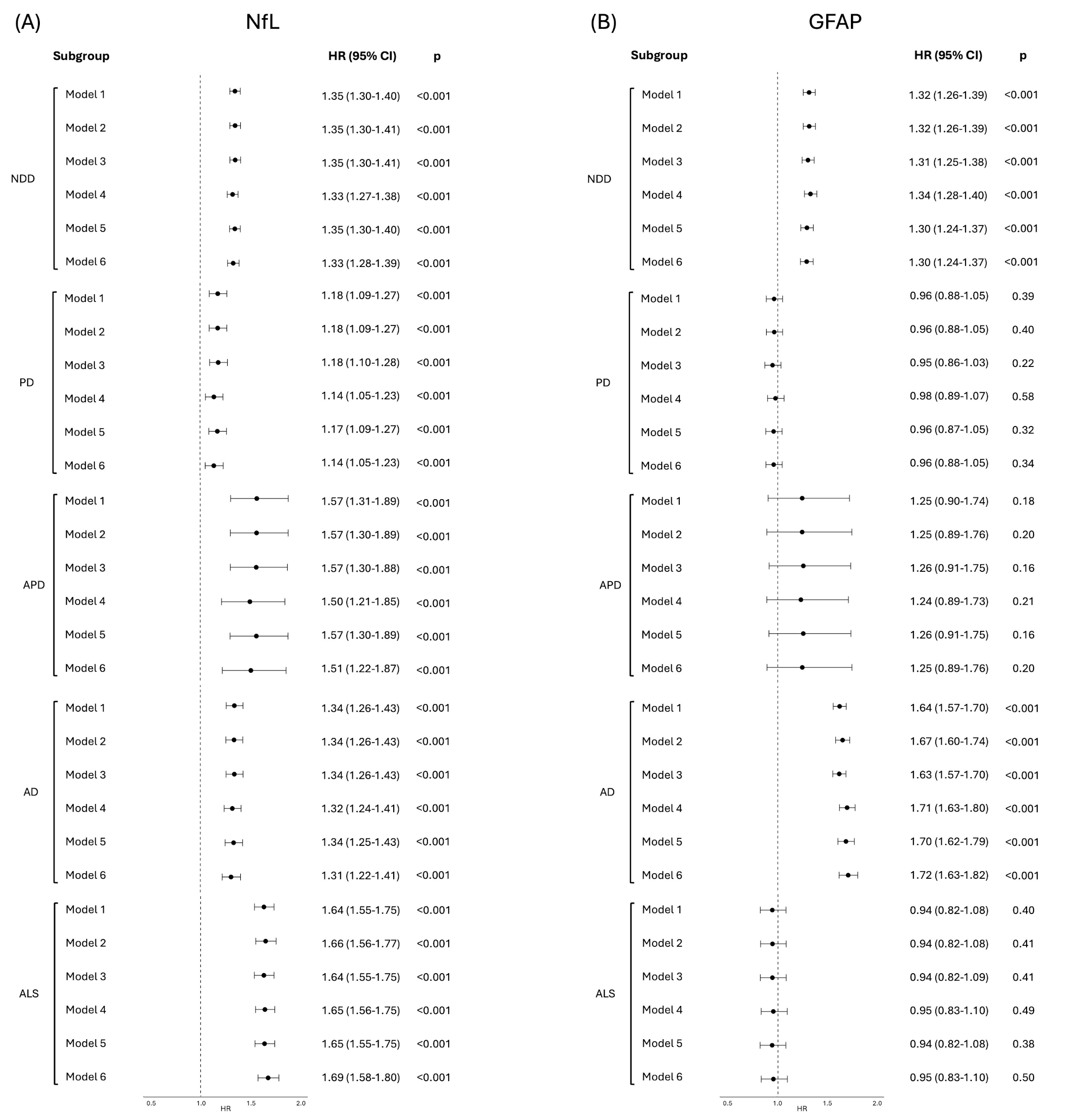
**Supplementary Figure 2.** Hazard ratios for the independent association of NfL and GFAP with incident neurodegenerative diseases.

(A) Forest plot showing hazard ratios (HRs) and 95% confidence intervals (CIs) for NfL across six adjustment models in any one of the neurodegenerative diseases of interest, as well as each diagnosis separately. (B) Forest plot showing the corresponding HRs and CIs for GFAP across the same models and diagnostic groups. Each HR estimate was derived from Cox proportional hazards models, adjusted for relevant covariates as described in the Supplementary Materials. The dashed vertical line is at HR = 1 (no association). *Abbreviations: HR = hazard ratio; CI = confidence interval; NfL = neurofilament light chain; GFAP = glial fibrillary acidic protein; NDD = neurodegenerative diseases; PD = Parkinson’s disease; APD = atypical parkinsonian disorders; AD = Alzheimer’s disease; ALS = amyotrophic lateral sclerosis.*

**Supplementary References:**

1. Inker LA, Eneanya ND, Coresh J, et al. New Creatinine- and Cystatin C-Based Equations to Estimate GFR without Race. N Engl J Med. 2021 Nov 4;385(19):1737-1749. doi: 10.1056/NEJMoa2102953.
